# Common deficiencies found in generic Finished Pharmaceutical Product (FPP) applications submitted for registration to the South African Health Products Regulatory Authority (SAHPRA)

**DOI:** 10.1186/s40545-021-00398-5

**Published:** 2022-01-12

**Authors:** Lerato Moeti, Madira Litedu, Jacques Joubert

**Affiliations:** 1 South African Health Products Regulatory Authority (SAHPRA), Pretoria, South Africa; 2 School of Pharmacy, University of the Western Cape, Cape Town, South Africa

**Keywords:** Finished Pharmaceutical Product (FPP), Common deficiencies, South African Health Products Regulatory Authority (SAHPRA), Non-sterile products, Sterile products

## Abstract

**Background:**

The aim of the study was to investigate the common deficiencies observed in the Finished Pharmaceutical Product (FPP) section of generic product applications submitted to SAHPRA. The study was conducted retrospectively over a 7-year period (2011–2017) for products that were finalised by the Pharmaceutical and Analytical pre-registration Unit.

**Methods:**

There were 3148 finalised products in 2011–2017, 667 of which were sterile while 2089 were non-sterile. In order to attain a representative sample for the study, statistical sampling was conducted. Sample size was obtained using the statistical tables found in literature and confirmed by a sample size calculation with a 95% confidence level. The selection of the products was according to the therapeutic category using the multi-stage sampling method called stratified-systematic sampling. This resulted in the selection of 325 applications for non-sterile products and 244 applications for sterile products. Subsequently, all the deficiencies were collected and categorised according to Common Technical Document (CTD) subsections of the FPP section (3.2.P).

**Results:**

A total of 3253 deficiencies were collected from 325 non-sterile applications while 2742 deficiencies were collected from 244 sterile applications. The most common deficiencies in the FPP section for non-sterile products were on the following sections: Specifications (15%), Description and Composition (14%), Description of the Manufacturing Process (13%), Stability Data (7.6%) and the Container Closure System (7.3%). The deficiencies applicable to the sterile products were quantified and the subsection, Validation and/or Evaluation (18%) has the most deficiencies. Comparison of the deficiencies with those reported by other agencies such as the USFDA, EMA, TFDA and WHOPQTm are discussed with similarities outlined.

**Conclusions:**

The overall top five most common deficiencies observed by SAHPRA were extensively discussed for the generic products. The findings provide an overview on the submissions and regulatory considerations for generic applications in South Africa, which is useful for FPP manufacturers in the compilation of their dossiers and will assist in accelerating the registration process.

## Background

Pharmaceutical companies use data accumulated during discovery and development stages of a pharmaceutical product in order to register and thus market the medicine. Throughout the development stages, they are required to abide by an array of strict rules and guidelines in order to ensure safety, quality and efficacy of the Finished Pharmaceutical Product (FPP) in humans [1]. Inspection of manufacturing plants and laboratory quality control analysis only do not guarantee product quality and safety [2]. All processes involved in the manufacture of the Active Pharmaceutical Ingredients (APIs) and the FPP need to be controlled [2]. Therefore, assessment of the product dossier prior to its acceptance is paramount [2]. Countries possess their own regulatory authority, which is responsible for enforcing the rules and regulations and issue the guidelines to regulate FPP development process, licensing, registration, manufacturing, marketing, labelling and the product life cycle of the FPP. In this highly regulated environment, regulatory affairs play a critical role as the leading department to provide strategic advice on extremely difficult decisions through the life of the FPP [1]. Even with the strict rules and guidelines, very few pharmaceutical companies submit quality dossiers which do not require any additional amendment or additions at initial review. Dossiers possessing a large number of deficiencies will necessitate more interaction between the authority and the manufacturer during the assessment process, thus increasing the turnaround times for registration of medicines [3]. Subsequently delaying patient access to urgently needed medication.

Over the years, a number of regulatory authorities have witnessed and reported on recurring deficiencies observed from the submitted dossiers. Authorities such as United States Food and Drug Administration (USFDA), European Medicines Agency (EMA) and Taiwan Food and Drug Administration (TFDA) have noted how the publication of common deficiencies has resulted in the submission of improved quality dossiers from pharmaceutical companies. The USFDA published a 4-part series citing the common deficiencies observed from the Abbreviated New Drug Applications (ANDA) on the quality aspects of the dossier. Part 1 of the series, dealt with the deficiencies cited in the API section [4]. Part 2–4 of the series was on common deficiencies observed from the FPP part of the dossier [5–7]. The 4-part series was however only qualitative and not quantitative. The TFDA also reported on common deficiencies witnessed in the FPP for applications submitted from June 2011 to the end of May 2012 [8], while the EMA’s study focused on applications finalised during the Committee for Medicinal Products for Human Use (CHMP), during 12 consecutive plenary meetings held between 2007 and 2008 [9]. The World Health Organization Pre-Qualification Team (WHOPQTm) reported on the deficiencies observed in the API and FPP sections for products submitted between April 2007 and December 2010 [3]. A guidance document was also published by the WHOPQTm in 2018 to alert manufacturers of the FPP deficiencies witnessed [10]. The studies conducted were aimed at collecting and analysing the quality review issues, which will serve as a reference and a communication medium for applicants to understand the regulatory requirements in the respective countries, which could be useful for compilation of the dossier and to facilitate the approval process.

South African Health Products Regulatory Authority (SAHPRA) has not implemented this transparency since the inception of the authority in 1965. The registration process by SAHPRA involves a scientific evaluation of the dossier submitted by the applicant in the form of a Common Technical Document (CTD). During this evaluation, a list of recommendations is generated related to the quality, safety and efficacy, which are forwarded to the applicant once discussed at the Pharmaceutical and Analytical (P&A) Committee meetings, to be addressed and resolved prior to approval. The P&A Committee managed to conclude and finalise on the scientific assessments of 3148 applications between 2011 and 2017. With SAHPRA receiving approximately 1200 applications annually, by 2016, a backlog of 7902 applications was accumulated. Within the period 2010–2015 only 3779 application were registered or rejected. From the backlog of applications, 4397 applications had not yet been allocated for evaluation while 3505 were in-process in the pre-registration phase. This shows the urgent need to employ measures such as collecting and analysing the quality review issues, thereby accelerating the approval process by the authority.

In order to identify general trends in the quality deficiencies for SAHPRA, we analysed all deficiencies from products finalised during the P&A Committee meetings over a 7-year period (2011–2017). The 3148 applications finalised during this period were considered a large sample to use for the study therefore a statistical sampling approach was employed to obtain a representative sample.

The manufacturing of the FPP is governed by precise requirements and guidelines such as good manufacturing practises and International Conference of Harmonisation guideline, ICH 3QB [11]. This is to ensure that the medicinal products are fit for their intended use and do not pose risks to the patients as a result of inadequate safety, quality or efficacy [12–14]. In the assessment of the medicines for registration by regulatory authorities, deficiencies are frequently observed in the applications, thus a proactive approach is intended in order to promote transparency between SAHPRA and the FPP manufacturers. The investigation undertaken is therefore aimed at identifying common deficiencies in the FPP section of applications submitted to SAHPRA. Publication of these will assist in the submission of quality dossiers which will accelerate the registration process and promote access to medicines for patients.

## Methods

**Fig. 1:**
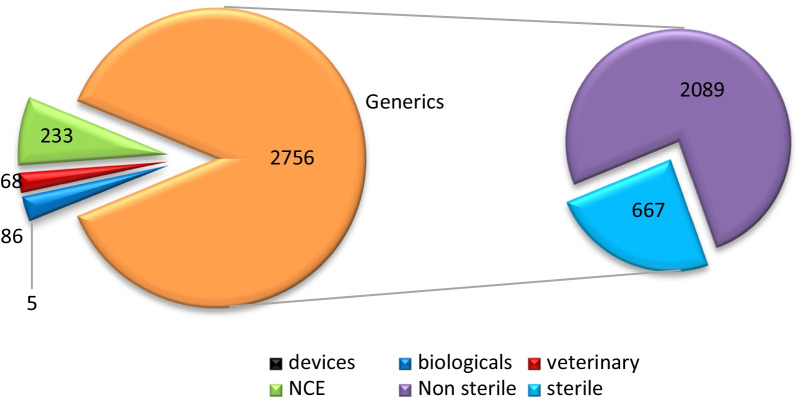
The distribution and grouping of the finalised products between 2011 and 2017 by the SAHPRA P&A Committee, pre-registration Unit

Overall 3148 applications were finalised in the 7-year period, of which 2089 were non-sterile products while 667 were sterile products. Veterinary (68), Biologicals (86), Medical Devices (5) and New Chemical Entities (NCEs) (233) were also finalised by the P&A Committee in the period as shown in Fig. 1, but was not included as part of this study. The NCEs were not included because they involve a more extensive evaluation, which required the compulsory submission of the restricted part of the Active Pharmaceutical Ingredient Master File (APIMF). As a result, a set of additional recommendations which are not observed in the generic applications is usually communicated to the applicant. Biologicals were not included due to the same reasons as the NCEs, as well as due to differences in the nature and preparation of the APIs used, this will necessitate a separate study as per the work published by the EMA on Biosimilars [15]. Veterinary products were not included since the P&A Committee was only providing support to the Veterinary Unit and each application requires the submission of Clinical trial data assessed by the Veterinary Clinical Committee, therefore it would be out of the scope of the research study. Lastly, Medical Devices were not included since the sample was too small to render the deficiencies as common. One of the main reasons for exclusively conducting a study for generics is that the generic applications constitutes majority of the applications received by SAHPRA annually and the lessons learnt from the generic products can also be employed for non-generic applications.

Given the large size of the submitted applications, a statistical method was applied to yield a representative sample adequate to use for the study. The calculated sample size obtained was 325 for the non-sterile products and 244 for the sterile products using the equations reported by Israel (1992) [16] and Kadam et al. (2010) [17] as Eqs. 1 to 4:

1 $$n_{0} = \frac{{Z^{2 } pq}}{{e^{2} }},$$

2 $$n = \frac{n.}{{1 + \frac{n. - 1}{N}}}.$$

The equations consist of the following parameters: *z* = the confidence level corresponds to a *z*-score, for a 95% confidence level *z* is 1.96. *p* = the degree of variability, *q* relates to degree of variability above, indicated as 1 − *p* depending on the variability of the population, *e* = level of precision which is ± 5% for the selected confidence level of 95%, *n*_0_ = sample size, *n* = adjusted sample size for population sizes that are less than 3000, and *N* = population size [17, 18].

Calculation for the sterile products is stipulated below with a population of 667. The same was applied for non-sterile products with a population of 2089 where the sample size of 325 was obtained:

3 $$\begin{aligned} n_{0} & = \frac{{Z^{2 } pq}}{{e^{2} }} \\ & = \frac{{1.96^{2} 0.5^{2} }}{{0.05^{2} }} \\ & = 384.16, \\ \end{aligned}$$

4 $$\begin{aligned} n & = \frac{n.}{{1 + \frac{n. - 1}{N}}} \\ & \frac{384.16}{{1 + \frac{384.16 - 1}{{667}}}} \\ n \, & = \, 244. \\ \end{aligned}$$

Comparison of the calculated sample size with the table reported by Mohammad [18] for a given population size showed similarity in that the reported value for a population of 650 is 242 with the same confidence interval and level of precision. There are many other tables reported [19–21] with sample size ranging between 240 and 255.

A multi-stage sampling method called stratified-systematic sampling was employed. In this method, the entire population is divided into a number of homogeneous groups usually known as “strata” and thereafter units are systematically sampled from each of these stratums [21].

It is pivotal to ensure that the selection is not random and biased. Stratified systematic sampling allows for this as it ensures that all critical variables are considered. Aspects such as the applicant, the dosage form, the API used, the therapeutic category and finalisation time of the drug product were considered as important variables when sampling is conducted. Out of the above five variables, the most critical is the therapeutic category since we are dealing with pharmaceutical products. The best way to categorise the products is through their therapeutic indications, i.e. function and pharmacological classification of the drug.

Regulation 25 of Act 101 classifies and categorise medicines in South Africa as follows:

- Category A for medicines which are intended for use in humans and which are, without manipulation, ready for administration, including packaged preparations where only a vehicle is added to the effective medicine;
- Category B for medicines which cannot be administered without further manipulation; and
- Category C for medicines intended for veterinary use, which are without further manipulation, ready for administration including packaged preparations where only a vehicle is added to the effective medicine [22].

All medicines in the population are category A. This category is subdivided into 34 pharmacological classifications, some of which are subdivided further. Each therapeutic category is considered a stratum. These are grouped into 19 categories as depicted in Table 1. The sample size in each stratum varies according to the relative importance of the stratum in the population, i.e. percentage contribution. For example, if 16% of the population are antiviral agents, then 16% of the sample should contain drug products in that group. From Table 1, each stratum is now treated as a population with a specific sample size. The strata are arranged in terms of therapeutic category of the applications. Thus, the numbers in the first column Table 1 are the number of finalised applications within that therapeutic category for sterile products. For example, there were 138 applications finalised with a pharmacological classification, central nervous system depressants.

**Table 1:** The different strata (pharmacological classifications) generated for sample selection of sterile products

Pharmacological classification (therapeutic categories)	Population (*N*)	%	Sample (*n*)
Central nervous system depressants	138	21	52
2.1 Anaesthetics			
2.2 Sedatives, hypnotics			
2.5 Anticonvulsants, including anti-epileptics			
2.7 Anti-pyretic or anti-pyretic and anti-inflammatory analgesics			
2.8 Analgesic combinations			
2.9 Other analgesics			
3.2 Non-hormonal preparations	12	1.8	4
4.0 Local anaesthetics	22	3.3	8
Medicines affecting autonomic function			
5.2 Adrenolytics (sympathicolytics)	62	9.3	23
5.4.1 Anti-Parkinson’s preparations			
5.7.1 Anti-histaminics			
5.7.2 Anti-emetics and anti-vertigo preparations			
5.10 Serotonin antagonists			
Vasodilators, hypotensive medicines			
7.2 Vasoconstrictors, pressor medicines	33	5.0	12
7.10.3 Other hypotensives			
Medicines acting on blood and haemopoietic system			
8.1 Coagulants, haemostatics	28	4.2	10
8.2 Anticoagulants			
8.3 Erythropoietics (haematinics)			
8.4 Plasma expanders			
Medicines acting on respiratory system			
10.2.1 Inhalants	6	1.0	2
Medicines acting on gastro-intestinal tract			
11.4.3, Antacids, other	10	1.5	4
Ophthalmic preparations			
15.4 Ophthalmic preparations. other	32	4.8	12
Medicines acting on muscular system			
17.1 Peripherally acting muscle relaxants	12	1.8	4
Medicines acting on genito-urinary system			
18.1 Diuretics	29	4.3	10
18.3 Ion-exchange preparations			
18.7 Contraceptive preparations	14	2.1	5
19.0 Oxytocics	22	3.3	8
Antibiotics and antibiotic combinations			
20.1.1 Broad and medium spectrum antibiotics	99	15	37
20.1.2 Penicillins			
20.2.2 Fungicides			
20.2.3 Tuberculostatics			
20.2.8, Antiviral agents			
Hormones, antihormones and oral hypoglycaemics			
21.1 Insulin preparations	59	8.9	22
21.2 Oral hypoglycaemics			
21.4 Parathyroid preparations			
21.5 Cortico-steroids			
21.10 Trophic hormones			
21.12 Hormone inhibitors			
26.0 Cytostatic agents	61	9.0	22
28.0 Contrast media	12	1.8	4
32.15 Radiopharmaceuticals	2	0.3	1
34, Other	14	2.1	5
	667	100	245

The *k*th term serves as a constant value used for systematic sampling and is calculated as illustrated in Eq. 5 with *N* as the population size and *n* as the calculated sample size [16]. A systematic sample would select the first element and thereafter the *k*th term on the list afterwards until the required sample has been selected in the whole population. The interval between the selected elements would then be the population size/calculated sample size [16]. The calculated *k*th term gave the value 2.7.3 (Eq. 6). This therefore makes the value three the *k*th term for the systematic sampling, i.e. in all strata. This resulted in the sample size of 245. However, 244 was used in accordance to the calculation using Eq. 2. Similarly, this was conducted for the non-sterile products to select the sample size of 325:

5 $$n = \frac{N}{{k{\text{th}}}},$$

6 $$k{\text{th}} = \frac{N}{{n}} = \frac{667}{{244}} = 2.73.$$

The full history of all products finalised in the 7-year period (2011–2017) were collected. The history comprises all communication between the authority and applicants in order to reach finalisation. The documents include the recommendations sent to the applicant and the responses received, as well as the evaluation reports of responses in the form of amendment schedules. These paper documents were obtained from the committee meeting minutes and the registry files where all documents relating to the product are placed. The investigation process involved obtaining the type and extent of the deficiencies raised in the first deficiency letter following the initial evaluation process, thereafter, extracting all the responses and feedback during multiple rounds of communication. During collection of the deficiencies, those with a frequency that was observed as less than five were categorised under “other” in the tables and calculated in the relevant section or subsection. The understanding was that these would not be classified as common due to the low frequency.

The study focuses mainly on the FPP which is presented as Module 3.2.P part of the CTD structure of the dossier as stipulated in Table 2, Module 3.2.P entails eight sections in which five consists of subsections. The 3.2.P sections are applicable for all types of medicines including sterile and non-sterile products.

**Table 2:** FPP (3.2.P) sections and subsections for classification of observations

CTD sections and subsections	Content
3.2.P.1	Description and Composition
3.2.P.2	Pharmaceutical Development
3.2.P.2.1	Components of the Pharmaceutical Product
3.2.P.2.2	Final Pharmaceutical Product
3.2.P.2.3	Manufacturing Process Development
3.2.P.2.4	Container Closure System
3.2.P.2.5	Microbial Attributes
3.2.P.2.6	Compatibility
3.2.P.3	Manufacture
3.2.P.3.1	Manufacturer(s)
3.2.P.3.2	Batch Formula
3.2.P.3.3	Description of Manufacturing Process and Process Control
3.2.P.3.4	Control of Critical Steps and Intermediates
3.2.P.3.5	Process Validation and/or Evaluation
3.2.P.4	Control of Inactive Pharmaceutical Ingredients
3.2.P.4.1	Specifications
3.2.P.4.2	Analytical Procedures
3.2.P.4.3	Validation of Analytical Procedures
3.2.P.4.4	Justification of Specifications
3.2.P.4.5	Excipients of Human Origin
3.2.P.4.6	Novel Excipients
3.2.P.5	Control of Finished Pharmaceutical Product
3.2.P.5.1	Specifications
3.2.P.5.2	Analytical Procedures
3.2.P.5.3	Validation of Analytical Procedures
3.2.P.5.4	Batch Analysis
3.2.P.5.5	Characterisation of Impurities
3.2.P.5.6	Justification of Specifications
3.2.P.6	Reference Standard or Materials
3.2.P.7	Container Closure System
3.2.P.8	Stability
3.2.P.8.1	Stability Summary and Conclusions
3.2.P.8.2	Post-approval Stability Protocol and Stability Commitment
3.2.P.8.3	Stability Data

The deficiencies obtained were reviewed and the frequency of each listed per section and subsection in 3.2.P together with the percentage frequency of the total deficiencies per section and subsection of the CTD, were calculated as follows:

- Percentage frequency of deficiency identified per section = (frequency of specific deficiency/total number of deficiencies per section of CTD) × 100.
- Percentage frequency of deficiency identified per overall 3.2.P = (frequency of specific deficiency/total number of deficiencies per overall 3.2.P section of CTD) × 100.

The deficiencies were collected and illustrated as charts and graphs using Microsoft Office Excel^®^ 2016 (Microsoft Corporation, USA).

## Results

### Deficiencies from non-sterile products

**Fig. 2:**
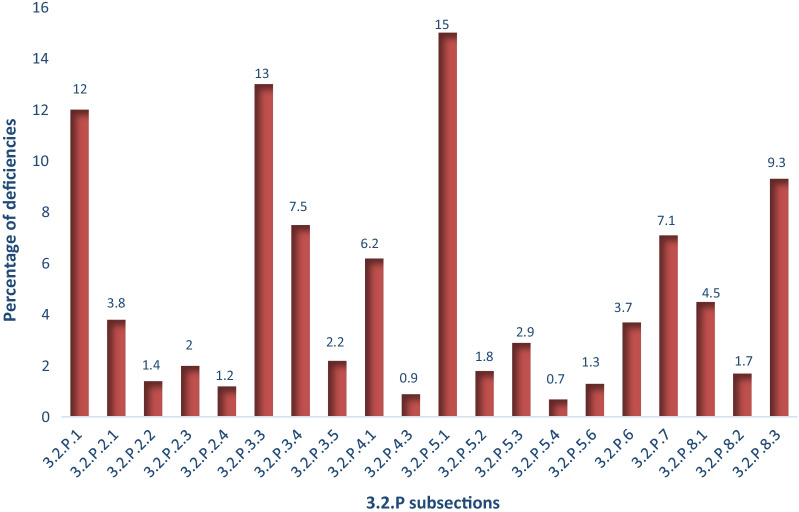
The distribution of all deficiencies found in the 3.2.P sections and subsections for non-sterile applications submitted to SAHPRA. Modules: 3.2.P.1 Description and Composition, 3.2.P.2.2 Final Pharmaceutical Product, 3.2.P.2.3 Manufacturing Process Development, 3.2.P.2.4 Container Closure System, 3.2.P.3.3 Description of the Manufacturing Process, 3.2.P.3.4 Control of Critical Steps and Intermediates, 3.2.P.3.5 Process Validation and/or Evaluation, 3.2.P.4.1 Specifications of IPIs, 3.2.P.4.3 Validation of Analytical Procedures of IPIs, 3.2.P.5.1 Specifications of the FPP, 3.2.P.5.3 Validation of Analytical Procedures of FPP, 3.2.P.5.4 Batch Analysis of the FPP, 3.2.P.5.6 Justification of Specifications, 3.2.P.6 Reference Materials, 3.2.P.7 Container Closure System, 3.2.P.8.1 Stability Summary and Conclusions, 3.2.P.8.2 Post Approval Stability Protocol and Stability Commitment, 3.2.P.8.3 Stability Data

The 325 applications contained a variety of dosage forms which are: film-coated and uncoated immediate release tablets (48%), immediate release capsules (23%), orodispersible tablets (8.0%), extended release tablets (8.0%), extended release capsules (3.5%), chewable tablets (1.2%), powders for suspensions (5.1%) and other (3.2%). The dosage forms which fall under the “other” category included oral solutions, creams, nasal spray, immediate release granules, gels, ointments, suppositories, lozenges and nose drops. A total of 3253 FPP deficiencies were collected from the 325 deficiency letters. Table 3 shows all deficiencies observed from generic non-sterile products that were finalised in the 2011–2017 period by the P&A pre-registration Unit. Figure 2 shows the distribution of the deficiencies and further highlights the 3.2.P sections in the CTD with the most deficiencies. The sections with the highest deficiencies are Module 3.2.P.3 Manufacture of the FPP, (23%) followed by Module 3.2.P.5 Control of the FPP (21%) and 3.2.P.8 Stability (15%). These three sections are considered the most critical sections in the CTD under Module 3.2.P as observed from reports on common deficiencies by other regulatory authorities while reporting [6–10].

**Table 3:** List of FPP common deficiencies in the 3.2.P section of the CTD recommended by SAHPRA for non-sterile products finalised by the pre-registration unit between 2011 and 2017

Subsection	Deficiency	Amount	% overall
3.2.P.1 Description and composition of the FPP			
3.2.P.1	Include an indication that water or other solvents are not present in the FPP since they have been eliminated during the manufacturing process	34	14
3.2.P.1	State the polymorphic form of the API(s) used in the unitary batch formula	52	
3.2.P.1	If a potency adjustment for the API has to be made, a statement to the effect that the actual quantity of the active will depend on the potency and the Pharmaceutical ingredients Inactive (IPI) that will be used to adjust the bulk quantity should be made. The manner in which the adjustment will be made should also be specified	48	
3.2.P.1	Include the grades of all the IPIs used in the formulation, or the functionality specification of the IPI, if applicable. Indication that it is a pharmaceutical grade is not sufficient	101	
3.2.P.1	The purpose of each IPI should be stated briefly. If the IPI is used for multiple purposes in the formulation, each purpose should be mentioned	31	
3.2.P.1	The Colour Index Numbers (Foodstuffs, Cosmetics and Disinfectants Act, 1972 Regulation Food Colourants) or the colourant reference number in accordance with the European directive of colourants for those used in the formulation	26	
3.2.P.1	The theoretical quantity of the base of the active pharmaceutical ingredient (API) should be stated if a compound, e.g., hydrate, solvate, salt is used	19	
3.2.P.1	The description of the FPP (including scoring) is incomplete and does not concur with other relevant sections in the dossier such as 3.2.P.5.1 and Module 1.3	32	
3.2.P.1	The theoretical mass must be indicated for uncoated tablets. In the case of coated dosage forms, the theoretical mass of the core, coating material, as well as the total mass of the dosage form/unit should be indicated	48	
3.2.P.1	Fill mass, type of gelatine used as well as the capsule size, composition and mass of the capsule should be indicated	21	
3.2.P.1	The overage used for the active pharmaceutical ingredient (API) should be indicated as a footnote and justified in 3.2.P.2.2	12	
	Other	19	
		443	
3.2.P.2 Pharmaceutical development			
3.2.P.2.1 Components of the pharmaceutical product			
3.2.P.2.1	A Pharmaceutical Development Report (generally of not more than 25 A4 pages) should be submitted with each application	13	3.8
3.2.P.2.1	Provide a brief summary of the synthesis of the API including a brief discussion of the physico-chemical characteristics of the API which are relevant to the final product	23	
3.2.P.2.1	Include a discussion of the stability of the final product formulation and conclusion on stability and shelf-life allocation in accordance with the P&A CTD guideline	10	
3.2.P.2.1	Explain the difference in specific excipients between the test and reference product	11	
3.2.P.2.1	Submit the compatibility studies of the API-IPI used in the formulation to confirm that these are compatible with each other	23	
3.2.P.2.1	Results from comparative in vitro studies (e.g., dissolution) or comparative in vivo studies (e.g., bioequivalence) should be discussed	45	
3.2.P.2.2 Final pharmaceutical product			
3.2.P.2.2	The reason for the overage should be stated/justified, e.g., with reference to batch results, in 3.2.P.2.2.2	21	1.4
3.2.P.2.2	Justify the choice and quantity of excipients used in the formulation	23	
3.2.P.2.3 Manufacturing process development			
3.2.P.2.3	The discriminatory nature of the selected dissolution medium should be illustrated	32	2.0
3.2.P.2.3	Provide justification of the selected dissolution quality control (QC) medium with the inclusion of a surfactant	34	
3.2.P.2.4 Container closure system			
3.2.P.2.4	Submit the discussion on the suitability of the formulation with the primary packaging system to confirm the acceptability of the proposed primary packaging	34	1.2
	Other	5	
		274	
3.2.P.3 Manufacture of the FPP			
3.2.P.3.3 Description of manufacturing process and process controls			
3.2.P.3.3	The description of the manufacturing procedure must include duration of treatment, manufacturing conditions (temperature and humidity) and specifications for machine settings and capacity	83	13
3.2.P.3.3	No provision has been made to bulk storage before packaging. Indicate the nature of the containers and maximum period the core and/or film-coated tablets may be stored (bulk) before final packaging. Submit information and provide supporting data with regard to holding time studies. This includes bulk holding time for cores prior to coating as well as container used	97	
3.2.P.3.3	The manufacturing process flowchart is inadequate, include the in-process controls, hold times for processing steps and other additional controls to ensure completeness	23	
3.2.P.3.3	The proposed holding times for intermediate products should to be included in the calculation of the shelf-life; they should not exceed 25% of the shelf life and if more than 30 days stability data should be submitted	29	
3.2.P.3.3	Describe the tablet compression procedure and compression speed included as well as coating parameters used	7	
3.2.P.3.3	The leak test, sealing test and adhesiveness for the blister packs must be described	11	
3.2.P.3.3	Drying time must be indicated and moisture content to which the granules are dried must be stated	24	
3.2.P.3.3	State the sieve sizes and mixing/blending speed during manufacture of the product as well as duration of stirring and drying temperature	76	
3.2.P.3.3	A brief description of the packaging procedure must be provided	33	
3.2.P.3.3	Fluid bed drying conditions must include inlet and outlet air temperature	6	
3.2.P.3.3	The manufacturing process outlined is inaccurate in comparison to the description and validation report	17	
3.2.P.3.4 Control of critical steps and intermediates			
3.2.P.3.4	The in-process control tests and frequency must be included as well as expansion of specifications for the granulate to include moisture content	88	7.5
3.2.P.3.4	Specification for uniformity of content of the divided tablet must be included and blend uniformity as an in-process test	41	
3.2.P.3.4	The limit for tablet hardness must be included as an in-process test and limits should be expressed in Newton and inclusion of the friability test	43	
3.2.P.3.4	Include the test for friability for uncoated tablets as an in-process control or in the final specifications	24	
3.2.P.3.4	Confirm that Batch Manufacturing records and packaging documents will be available upon request or during inspection	10	
3.2.P.3.4	Limits proposed on the critical steps were not accepted and further justification is required	32	
	Other	6	
3.2.P.3.5 Process validation and/or evaluation			
3.2.P.3.5	Submit a bulk formula for each batch size for each strength as three master manufacturing batch records were submitted with different batch sizes	4	2.2
3.2.P.3.5	Include validation report for three commercial batches to confirm reproducibility and batch to batch consistency of the manufacturing process	43	
3.2.P.3.5	Provide validation protocol and/or report for the proposed batch size	25	
		722	
3.2.P.4 Control of inactive pharmaceutical ingredients			
3.2.P.4.1 Specifications			
3.2.P.4.1	Quantitative and qualitative composition of the colourant must be included	26	6.2
3.2.P.4.1	Provide a declaration that the IPI, e.g., talc is asbestos free	7	
3.2.P.4.1	Submit the certificate of analysis for each of the IPIs used	32	
3.2.P.4.1	Include specifications and control procedures of the IPIs used in the formulation for non-pharmacopoeial	32	
3.2.P.4.1	Provide evidence that the IPIs are transmissible spongiform encephalopathies/bovine spongiform encephalopathies (TSE/BSE) free	44	
3.2.P.4.1	The related substances controlled in the IPIs should be quantified	45	
3.2.P.4.1	Provide the identification used for the colourant or dye, for example a UV spectrum	16	
3.2.P.4.1	Confirm that the colourant complies with purity criteria of the Foodstuffs, Cosmetics and Disinfectants Act, Act 54 of 1972 or with directives of the European countries or the register of the USFDA	32	
3.2.P.4.3 Validation of analytical procedures			
3.2.P.4.3	Validation data were not submitted for analytical testing methods of non-pharmacopoeial substances. Submit	16	0.9
	Other	13	
		263	
3.2.P.5 Control of FPP			
3.2.P.5.1 Specifications			
3.2.P.5.1	The dissolution specification must be brought in line with the profiles of the biostudy and reference products for this parameter. All the strengths of both test and reference products demonstrated very rapid dissolution whereas the specification is not in line with the definition of rapid dissolution	139	15
3.2.P.5.1	The dissolution specification for release and shelf-life must correspond	16	
3.2.P.5.1	Tighten the assay release and stability specification to 95–105% in accordance with the PA guidelines and include this as a percentage label claim	80	
3.2.P.5.1	The final product specification must be expanded to include a limit for residual solvents and the relevant validated control procedure must be described	16	
3.2.P.5.1	The FPP specifications should include an additional identification test	23	
3.2.P.5.1	Include the leak test to confirm that the product is protected from moisture in the final FPP specifications or as an in-process control	11	
3.2.P.5.1	Include all the parameters to be controlled for the Final product, i.e. FPP specifications at release and shelf life	9	
3.2.P.5.1	Tighten the specifications for water content taking into consideration the increased formation of impurities by water hydrolysis and the fact that the stability results do not justify the proposed specification	22	
3.2.P.5.1	Include authorised documentation code and date of authorisation for release and stability specifications (version control)	19	
3.2.P.5.1	Bring the degradation/related impurity limits of the FPP in line with the ICH guideline Q3B	16	
3.2.P.5.1	Tighten specifications for Total impurities to be in line with the stability and batch analyses results	48	
3.2.P.5.1	Tighten the shelf life specification limits of the specified and unspecified impurities, as they appear to be wider	45	
3.2.P.5.1	Tighten specifications for disintegration time since the final product is highly soluble	11	
3.2.P.5.1	Include a test for microbial purity in the FPP specifications	9	
3.2.P.5.1	Bring the FPP specifications in line with those indicated in a recognised pharmacopoeial monograph	15	
3.2.P.5.2 Analytical procedures			
3.2.P.5.2	The pore size of the filter must be stated in the dissolution method description or justified	21	1.8
3.2.P.5.2	Dissolution method should specify inline filtration or filtered immediately. The method for withdrawal and filtration of samples must ensure that dissolution of undissolved particles does not occur after sampling	38	
3.2.P.5.3 Validation of analytical procedures			
3.2.P.5.3	Submit validation data for the assay method of the API, residual solvents and related substances/degradation products	28	2.9
3.2.P.5.3	The following inconsistencies were observed in the submitted validation data which required clarification: nature of stress used in stress samples used in validation not confirmed, reference standard not calibrated against an internal standard; linearity of potency assay not conducted, detection limit for some specified related substances/residual solvents, acceptance criteria for system suitability tests and other parameters not justified	32	
3.2.P.5.3	Representative chromatograms should be submitted for validation of analytical methods	21	
3.2.P.5.3	Submit validation data of forced degradation studies in the assay method	12	
3.2.P.5.4 Batch analysis			
3.2.P.5.4	Submit a complete analysis data of at least two batches	23	0.7
3.2.P.5.6 Justification of specifications			
3.2.P.5.6	Justification of specifications was not submitted and requested	11	1.3
3.2.P.5.6	The proposed justification of specifications is inadequate and not accepted. An amendment is proposed in 3.2.P.5.1	21	
	Other	11	
		697	
3.2.P.6 Reference standard or materials			
3.2.P.6	Supply information on the primary reference standard used to confirm traceability if pharmacopoeial and describe how the secondary reference standards were established	19	3.7
3.2.P.6	Provide certificate of analysis (CoAs) of the reference standards used	32	
3.2.P.6	Provide the CoAs showing the results of the identification, purity and content of the reference standards used	43	
3.2.P.6	Characterisation of the reference and impurity reference standards not complete or inadequate	12	
	Other	14	
		120	
3.2.P.7 Container closure system of the FPP			
3.2.P.7	Include an identification test, e.g., IR of the immediate container closure system	31	7.1
3.2.P.7	Give a specification and demonstrate the integrity for the heat seal bond strength as well chemical nature and identification test for this heat seal lacquer in the aluminium foil	27	
3.2.P.7	Specify the printing details on blisters and give a control test for the quality of the printing	7	
3.2.P.7	The chemical nature of the desiccant must be disclosed	13	
3.2.P.7	Identification, chemical nature and density of the container closure must be included as well as specifications and the relevant control procedure included. This includes colour, dimensions and thickness	38	
3.2.P.7	The manufacturers of the primary packaging materials should be included	23	
3.2.P.7	Information included in the packaging insert/patient information leaflet (PI/PIL)/label is not in accordance with the packaging presentations contained in this section. Correct	21	
3.2.P.7	The certificates of analysis (CoAs) for the immediate container closure(s) used were not provided	43	
	Other	28	
		231	
3.2.P.8 Stability of the FPP			
3.2.P.8.1 Stability summary and conclusions			
3.2.P.8.1	Provide a justification for the out of trend assay results	28	4.5
3.2.P.8.1	The shelf-life specifications are incomplete or have missing criteria or parameters. Include these or provide a justification for not including the parameters listed in 3.2.P.5.1	32	
3.2.P.8.1	Indicate the date of initiation of the stability studies	15	
3.2.P.8.1	Include the minimum and maximum size of the batches placed under stability study	32	
3.2.P.8.1	Submit stability data for an alternative local packer for final products manufactured in a different country to the manufacturer, on the product packed in bulk containers over a suitable period covering the relevant transport conditions	29	
3.2.P.8.1	Indicate the type of batch, e.g., pilot/production/experimental as well as the batch size. For pilot batches, a provisional shelf life of up to 24 months is allocated	11	
3.2.P.8.2 Post-approval stability protocol and stability commitment			
3.2.P.8.2	The proposed post-approval stability study did not include the batches being placed on stability annually or how many batches per strength are annually put on stability testing	34	1.7
3.2.P.8.2	The proposed stability programme commitment is not in accordance with the stability guideline; Summary tables with test results from stability studies conducted under accelerated and stressed conditions were not submitted	21	
3.2.P.8.3 Stability data			
3.2.P.8.3	Correct the container closure system to correspond with that indicated in the container closure section, Module 3.2.P.7	36	9.3
3.2.P.8.3	Impurity/degradation shelf-life limits should be tightened from a quality perspective in view of the results observed for commercial batches	56	
3.2.P.8.3	Critical stability indicating parameters such as related substances and dissolution are not included in the stability testing. These should be included	54	
3.2.P.8.3	The proposed shelf life is not supported by the submitted studies, provide additional data to support the proposed shelf life, which should now be reasonably available	98	
3.2.P.8.3	Stability studies for different manufacturing sites were not provided, confirming similar stability. Submit	34	
3.2.P.8.3	Submit photostability data under normal conditions which show that secondary packaging protects the ultra-violet ray (UV)-sensitive API and that unrelated impurities did not increase with exposure to light and UV	14	
	Other	9	
		503

Table 3 specifies all the deficiencies observed in the 3.2.P section of the dossier. The deficiencies were calculated as percentage of the deficiencies in each subsection per overall 3.2.P section. For example, there were 274 deficiencies on the pharmaceutical development section, 3.2.P.2, which is granulated as 3.8% for 3.2.P.2.1 components of the pharmaceutical product, 1.4% for 3.2.P.2.2, final pharmaceutical product, 2.0% for 3.2.P.2.3, manufacturing process development and 1.2% for 3.2.P.2.4 container closure system for each subsection in the table.

The results in Table 3 are depicted as a chart in Fig. 2 to clearly show which subsection exhibits the highest and the lowest number of deficiencies. Subsection 3.2.P.5.1 has the highest deficiency covering 15% (71% of the 3.2.P.5 section). Module 3.2.P.1, Description and Composition of FPP, has the second largest number of deficiencies (14%). Module 3.2.P.3.3, Description of the Manufacturing Process has the third highest percentage of deficiencies (13%) with Module 3.2.P.8.3 on stability data of the FPP at 9.3% (66% of the 3.2.P.8 section).

### Deficiencies from sterile products

A similar investigation as for the non-sterile products was conducted for sterile products. The 244 sterile product applications consisted of the following dosage forms: concentrate for injection (35%), powder for injection (17%), lyophilised powder for injection or infusion (42%), ophthalmic solutions (4.8%), irrigation solution (0.8%) and a minority of other comprising the remaining 0.4%. These dosage forms were sterile suspensions and chelating agents. A total of 2742 FPP deficiencies related to sterile products were collected from 244 letters.

The 244 letters were obtained and deficiencies outlined in Table 4. Note that the CTD has different requirements in specific sections depending on the dosage form. For example, the sterilisation method selected for sterile products would need to be clearly indicated and justified in accordance to the decision trees for selection of the sterilisation methods (CPMP/QWP/054/98) [23] under 3.2.P.2.2. This is not a requirement for non-sterile products. There are a number of these sections in the CTD and those deficiencies are listed in Table 4. There are also a number of common sections where the requirements are the same whether a product is sterile or not, for example, 3.2.P.6 Reference Materials, 3.2.P.5.4, Batch Analysis, 3.2.P.5.5 Characterisation of Impurities, etc. Therefore, the deficiencies for sterile products are over and above those listed under Table 3 for non-sterile products depending on their applicability to the dosage form.

**Table 4:** List of FPP common deficiencies in the 3.2.P section of the CTD recommended by SAHPRA for sterile products finalised by the pre-registration Unit between 2011 and 2017

Section/subsection	Deficiency	Amount	% overall
3.2.P.1 Description and composition of the FPP			
3.2.P.1	Nitrogen is used as pressure source for filtration it must be indicated in the list of excipients and controlled in 3.2.P.5	74	3.1
	Other	12	
		86	
3.2.P.2 Pharmaceutical development			
3.2.P.2.2 Final pharmaceutical product			
3.2.P.2.2	The product development report is insufficient. It does not address the development of the buffered blend for filling, neither does it address aspects such as choice of container closure system, filter media, sterilisation methods	39	13
3.2.P.2.2	It is stated that sterile filtration is chosen as method of sterilisation without justification. The choice of sterilisation by filtration as the method of sterilisation must be scientifically justified in terms of the decision tree for sterilisation choices for aqueous products (CPMP/QWP/054/98). Terminal sterilisation should normally be the method of choice if the product is expected to be heat stable	106	
3.2.P.2.2	Discuss the selection and effectiveness of preservative	34	
3.2.P.2.2	Include the pore size of the filter used for the method of sterilisation	67	
3.2.P.2.2	The volume of overfills were unjustified in pharmaceutical development. Provide data to support that the indicated total fill volume sufficient to administer nominal dose	34	
3.2.P.2.2	Provide results of tests on extractable volume and the API content after reconstitution of the FPP with the selected solvent	76	
3.2.P.2.3 Manufacturing process development			
3.2.P.2.3	Justify sterilisation by filtration. Heat instability during autoclaving has been determined at 121 °C/20 min. Have studies been done at reduced F_o_ – values to confirm that terminal sterilisation is not possible	45	1.6
3.2.P.2.4 Container closure system			
3.2.P.2.4	Submit in-use stability testing method and results in this section to confirm integrity of the container closure system to prevent microbial contamination	32	1.9
3.2.P.2.4	The consistency for droplet size for the dropper used should be conducted to ensure that the same API/FPP is ejected at each drop	21	
3.2.P.2.6 Compatibility			
3.2.P.2.6	Extractability and leaching studies of the selected filter should be submitted	45	6.3
3.2.P.2.6	The studies to confirm the compatibility of the product with the recommended intravenous (IV) solutions was not conducted	54	
3.2.P.2.6	Provide compatibility studies of the formulation with the equipment used in the manufacturing process	31	
3.2.P.2.6	Compatibility and leaching studies of the formulation with the coated rubber stoppers to demonstrate that these do not cause leaching should be submitted	23	
	Other	19	
		626	
3.2.P.3 Manufacture of the FPP			
3.2.P.3.3 Description of manufacturing process and process controls			
3.2.P.3.3	The information must include an inspection flow diagram describing both processes, the batch manufacturing formulae, a comprehensive flow diagram and a comprehensive description detailing the various stages of both steps in the manufacturing process including environmental classification of areas, sterilisation methods and conditions of containers and equipment	54	13
3.2.P.3.3	Nitrogen is used as pressure source for filtration, it must be indicated in 3.2.P.3.3 and should be indicated in the formula and controlled in 3.2.P.5. In addition, the method of sterilisation used for nitrogen should be stated	43	
3.2.P.3.3	Confirm that the filter integrity is confirmed before and after filtration. Reference to the process procedure only to conduct filter integrity test is inadequate	23	
3.2.P.3.3	State the type and size (porosity) of the filters used for filtration of the solution	45	
3.2.P.3.3	Describe the grades of clean areas for manufacture and filling process of water for injection/diluent	82	
3.2.P.3.3	Provide lyophilisation conditions of the cycle used and confirm that the lyophiliser is sterilised after each cycle	68	
3.2.P.3.3	Proof of efficacy of the sterilisation of the dead space in the connecting tube and twist off ports of the bags must be provided	27	
3.2.P.3.4 Control of critical steps and intermediates			
3.2.P.3.4	Bioburden testing and the acceptance criteria for bioburden must be included as an in-process control measure	59	2.2
3.2.P.3.5 Process validation and/or evaluation			
3.2.P.3.5	Provide summary reports on the validations for the sterilisation of the rubber closures and for the lyophilised powder	76	17
3.2.P.3.5	The validation of sterilisation and depyrogenation processes with conditions and determination of maximum holding/processing times must also be included	83	
3.2.P.3.5	The hold time validation data should include hold time before and after filtration of final product bulk or hold time within lyophiliser chamber after cycle completion	34	
3.2.P.3.5	Provide summary reports on the validations of depyrogenation of the glass vials and sterilisation of the rubber closures and for the water for injection/diluent	23	
3.2.P.3.5	Submit a summary report of the validation (qualification) of the sterilisation cycle of the final product including the loading patterns	23	
3.2.P.3.5	Submit a summary report of the validation of the selected filter	16	
3.2.P.3.5	Provide a protocol or report of the validation of autoclaves and sterilisation/depyrogenation tunnels	23	
3.2.P.3.5	Provide a protocol or summary report of the media fill procedures and validation of holding times	43	
3.2.P.3.5	Include a summary report on autoclaving of production equipment	45	
3.2.P.3.5	A number of issues on the media fill validation including; Media fill validation not covering all product volumes and container types, details of the media fill conditions were not described, Aseptic process not validated by media fill to name a few	65	
3.2.P.3.5	The validation process should contain storage and shipping conditions linked to process validation results	25	
	Other	16	
		873	
3.2.P.4 Control of inactive pharmaceutical ingredients			
3.2.P.4.1 Specifications			
3.2.P.4.1	Nitrogen is used as pressure source for filtration. Provide specifications and control procedures	56	4.5
3.2.P.4.1	Indicate the leak test performed on the container closure system during filling	45	
	Other	23	
		124	
3.2.P.5 Control of FPP			
3.2.P.5.1 Specifications			
3.2.P.5.1	Seal integrity testing (leak testing) of ampoules must be included as a final product control	23	11
3.2.P.5.1	Visible particulate matter should be included as a specification either as final product release specification or as in-process control	54	
3.2.P.5.1	Bacterial endotoxin test (BET) should be included as a specification either as final product release specification or as an in-process control	80	
3.2.P.5.1	In view of the batch release data and stability data provided for related substances the justification of the specifications for total impurities based on batch release data is not accepted and should be reconsidered	34	
3.2.P.5.1	Include a specification for preservative effectiveness. The test is not required for routine analysis provided that the preservative effectiveness has been established at the lowest limit specified, however, the specification should be retained as a skip test	43	
3.2.P.5.1	The following were missing from the specifications and should be submitted: preservative efficiency testing at the end of shelf life; active content in reconstituted solution; product-related impurities in specifications considered as too wide; acceptance and extractable volume after reconstitution as well as uniformity of mass	22	
3.2.P.5.3 Validation of analytical procedures			
3.2.P.5.3	Provide validation data for the sterility test method. If a pharmacopoeial method from a recognised pharmacopoeia is used partial validation data will suffice	23	2.5
3.2.P.5.3	Provide validation data for the bacterial endotoxin test method	45	
3.2.P.5.6 Justification of specifications			
3.2.P.5.6	There were unjustified items: bacterial endotoxin limits; pH specification limits; active salt selection; omission of impurities in specifications and missing container closure test	54	2.8
	Other	22	
		400	
3.2.P.7 Container closure system of the FPP			
3.2.P.7	Consistency of the droplet size should be confirmed	45	7.2
3.2.P.7	Coating composition of the stoppers used was not included	27	
3.2.P.7	The CoAs for glass and rubber stoppers used were not provided	17	
3.2.P.7	Sterilisation of primary packaging components was not satisfactorily described	13	
3.2.P.7	Compatibility of the stopper material with the final product was not demonstrated on potential extractables. Extractability and leaching study is therefore requested	39	
3.2.P.7	Leachability study of the leachables originating from the container closure system should be investigated	34	
	Other	21	
		196	
3.2.P.8 Stability of the FPP			
3.2.P.8.3 Stability data			
3.2.P.8.3	Provide results of the stability studies on the diluted solution in selected diluent for infusion confirming the recommendations in the PI	28	13
3.2.P.8.3	The results of the photo stability studies showing no effect to impurity values and thus no requirement for protection from light during storage of the product should be provided	45	
3.2.P.8.3	The results of the in-use stability study confirming stability of the product at a specific temperature for specified amount of time as indicated in the PI and in accordance with the guidelines should be provided	38	
3.2.P.8.3	The results of the transportation stability test at specified elevated storage condition for a sufficient amount of time should be submitted	23	
3.2.P.8.3	Provide stability results to confirm the effectiveness of the preservative	43	
3.2.P.8.3	Stability studies should be conducted in upright and inverted positions, the results were only submitted for samples stored in an upright position. Submit for the inverted position	34	
3.2.P.8.3	There were missing tests during stability studies, for example, volume in container, sterility and BET. This should be conducted in the next testing and submitted	44	
3.2.P.8.3	Missing or insufficient data for aspects such as vacuum stress for container closure ingress testing; supporting storage out of Refrigeration; potency test performance during stability control; chromatograms from final product long-term, accelerated, and stressed stability studies and sterility tests on preservative efficiency	38	
3.2.P.8.3	Stability studies for temperature excursions at the end of the shelf-life should be submitted	36	
	Other	15	
		344	
3.2.R.1 Pharmaceutical and biological availability			
3.2.R.1*	Data to substantiate efficacy have been provided in Module 3.2.P.2 where essential similarity of the innovator and test product was proven however, a request for exemption from submitting proof of Biological availability based on the Biostudies Guidelines was not stipulated. Exemption will only be considered when motivation and comparative data have been submitted in Module 3.2.R.1	93	3.4
		93	

Note that there are deficiencies applicable to sterile products already included in Table 3, these were not included in this table to avoid duplication and quantified as other in the table due to the low frequency

*A regional requirement for sterile and liquid dosage form to request exemption from submitting proof of efficacy studies, only essential similarity with an SA innovator product is required in such cases

**Fig. 3:**
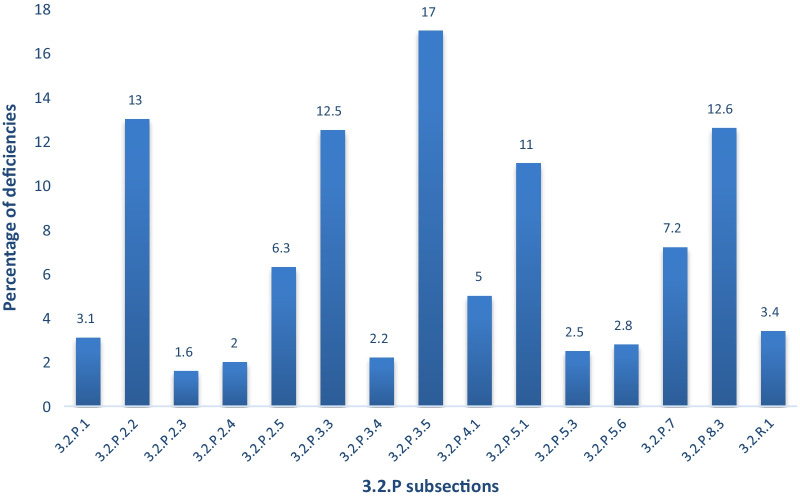
The distribution of deficiencies relating to sterile products. Modules: 3.2.P.1 Description and Composition, 3.2.P.2.2 Final Pharmaceutical Product, 3.2.P.2.3 Manufacturing Process Development, 3.2.P.2.4 Container Closure System, 3.2.P.2.5 Compatibility, 3.2.P.3.3 Description of the Manufacturing Process, 3.2.P.3.4 Control of Critical Steps and Intermediates, 3.2.P.3.5 Process Validation and/or Evaluation, 3.2.P.4.1 Specifications of IPIs, 3.2.P.5.1 Specifications of the FPP, 3.2.P.5.3 Validation of Analytical Procedures of FPP, 3.2.P.5.6 Justification of Specifications, 3.2.P.7 Container Closure System, 3.2.P.8.3 Stability Data, 3.2.R.1 Pharmaceutical and Biological Availability

Figure 3 highlights the most frequently observed deficiencies from the sterile products. It shows that FPP subsections Module 3.2.P.3.5, Process Validation and/or Evaluation (17%), Module 3.2.P.2.2, Development of FPP (13%), Module 3.2.P.8.3, Stability Data (12.6%), Module 3.2.P.3.3, Description of the Manufacturing Process (12.5%) and Module 3.2.P.5.1, Specifications (11%) fall under the top five most common deficiencies requested by SAHPRA for sterile products.

## Discussion

The most frequent common deficiencies observed by SAHPRA in the submitted non-sterile and sterile products are extensively discussed below as depicted Figs. 2 and 3.

### Deficiencies in Module 3.2.P.3., manufacture of the FPP

The highest section reported as per Fig. 2 was Module 3.2.P.3. Further analysis (Fig. 3) reveals that 13% of the overall deficiencies were due to Module 3.2.P.3.3—Description of Manufacturing Process and Process Control, 7.4% on Module 3.2.P.3.4—Control of Critical Steps and Intermediates and 2.2% on Module 3.2.P.3.5—Process Validation and/or Evaluation. Concerning sterile product deficiencies, a similar trend is witnessed where the highest reported section is Module 3.2.P.3, manufacture of the FPP. Module 3.2.P.3.5, Process Validation and/or Evaluation, constitutes 17% of the deficiencies, followed by 12.5% from Module 3.2.P.3.3, Description of the Manufacturing Process and lastly, 2.2% from Module 3.2.P.3.4, Control of Critical Steps and Intermediates.

The common deficiencies observed in the manufacturing process of non-sterile products included: insufficient information being provided on the manufacturing process such as duration of treatment; manufacturing conditions (temperature and humidity); specifications for machine settings; capacity of equipment compression procedure and speed; sieve sizes used; duration of stirring and drying temperatures. These and more are critical parameters that should be included in the process to provide the evaluator with a comprehensive description of the manufacturing process. The second deficiency was on the hold time period not being indicated as well as the bulk containers used for the intermediates and final product before packaging. The proposed holding time is dependent on the shelf life, whereby a holding time exceeding 25% of the shelf life [24] should be supported by accelerated and long-term stability data for approval. There were a large number of deficiencies where applicants did not indicate the proposed period, did not provide a hold time study report in Module 3.2.P.3.5, process validation and/or evaluation and supporting data in 3.2.P.8.3, stability data, if the proposed period exceeds the acceptable conditions as indicated above.

The common deficiencies witnessed from the sterile products in this prevalent section was on subsection, Module 3.2.P.3.5 Process Validation and/or Evaluation. The deficiencies included issues on the validation and outstanding summary report on validation of; the sterilisation method used, media fill procedures, depyrogenation of glass containers and sterilisation for rubber stoppers and autoclaving of production equipment. These are a requirement and should normally be submitted by the manufacturer when the product is considered sterile using aseptic processing or terminal sterilisation. It is imperative that the container used, the excipients, the FPP and container closures be sterile or sterilised for these products, therefore, summary reports on how the validation is conducted is vital. Media fill simulations are also of importance as they assess the performance of an aseptic manufacturing procedure using a sterile microbiological growth medium, in place of the FPP solution, to test whether the aseptic procedures are adequate to prevent contamination during actual FPP production [25–27]. The section comprised 54% of these deficiencies.

A common deficiency in the section, 3.2.P.3, Manufacture of the FPP, is the lack of inclusion of environmental classification of areas in the manufacture of sterile products. The classified rooms help the sterile pharmaceutical industry to manufacture products that are free from particulate and microbial contamination [27, 28]. The areas have a controlled contamination level, which is specified regarding the number of particles for every cubic meter for a specified particle size. These restricted areas are constructed with strict humidity, temperature and pressure control conditions to minimise the generation, introduction and retention of particulate matter inside the rooms [28, 29]. The classifications are either A, B, C and D with sterile environments normally using Class A or B or a combination of both. This requirement is therefore very critical in the manufacture of a sterile product and should be specified in the process. These deficiencies comprised 16% of the section.

### Deficiencies in Module 3.2.P.5., control of the FPP

The section with the second highest deficiencies is Module 3.2.P.5, control of the FPP, (21%) as depicted in Fig. 2. Figure 3 further shows that subsection 3.2.P.5.1, Specifications, had the most deficiencies in the whole 3.2.P reported for non-sterile products. Missing dissolution profiles and/or unacceptable dissolution limits were observed from nearly all the applications. Multimedia dissolution profile data on the biostudy test product is critical and used as reference data set that is used to support and assign dissolution limits in accordance to the EMA reflection paper [30]. The reports indicate that manufacturers often assign dissolution limits that are wider than the biostudy test product. This leads to back and forth communication between the applicant and the authority. Applicants often justify the widened limits based on the results of the stability results, however, this is not accepted since the acceptance criterion set should be based on the biostudy product. The behaviour should not change during stability as any deviation confirm deterioration of product quality. This is also part of the reason why the proposed dissolution specifications for release and shelf life should not differ as the product quality is expected to remain the same throughout shelf life as per the biostudy test product.

Module 3.2.P.5.1, Specifications, contains a number of deficiencies (58%) involving the request to tighten the proposed specifications based on batch analyses data, stability results and limits as indicated in ICH guidelines. For degradation/related impurities, manufacturers are required to ensure that the proposed specifications are in line with the recognised pharmacopoeia or that the limit is in accordance with ICH guidelines Q3B (R2) [11]. The limit should be below the calculated qualification threshold or reporting threshold. It was also observed that the acceptance criteria set for any other unknown impurities did not conform to ICH requirements. Impurities that are structural alerts for genotoxicity need to be controlled at the Threshold of Toxicological Concern (TTC) of 1.5 mcg/day, as found in the European Medicines Agency (EMA) [31, 32] and draft FDA guidance [33]. However, a higher limit may be proposed based on safety studies demonstrating that the proposed limit does not pose a safety concern. Other limits such as water content, assay, disintegration time are based on the batch analyses and stability results observed. A reasonable proposed limit would need to be justified by supporting data for acceptability if not already indicated in the pharmacopoeia or guidelines.

The most frequent deficiency observed for sterile products in this subsection is the request to include the limit for bacterial endotoxin in the FPP specifications. Endotoxins released from Gram-negative bacteria are the main reason of contamination in pharmaceutical products and as a result of this, an endotoxin test is required to be performed on sterile products especially those which are to be injected in the body so as to avoid bringing adverse effects to human [34].

### Deficiencies in Module 3.2.P.8, stability

The section with the third highest deficiencies is Module 3.2.P.8, Stability of the FPP, (15%) for non-sterile products. It comprises Module 3.2.P.8.1 (7.6%), -Stability Summary and Conclusions, Module 3.2.P.8.2 (1.8%) Post-Approval Stability Protocol and Stability Commitment and Module 3.2.P.8.3 (9.3%)—Stability Data. The frequent deficiencies in subsection 3.2.P.8.3, Stability Data, were on the limits proposed on degradation impurities and total impurities being too wide and applicant requested to tighten them in reference to the stability results, this relates to subsection 3.2.P.5.1, Specifications, as discussed above. The other deficiency was on the applicant omitting critical stability indicating parameters such as dissolution, total impurities or degradation impurities in the stability testing. Acceptance of a product cannot be granted if the stability testing does not include these critical parameters which determine the behaviour of the product throughout its shelf life.

There were 12.6% of the additional deficiencies specific to sterile products witnessed in subsection 3.2.P.8.3, Stability Data. The deficiencies were on the request for results of the in-use stability study confirming stability of the product at a specific temperature for a specified amount of time as indicated in the Professional Information (PI). Since the products are sterile, there is a requirement that if the product is not for single use such as ophthalmic solutions, lyophilised powders for infusion, etc., stability results should be conducted to confirm that the product quality is not compromised while in-use. Another list of stability data required involved studies to confirm compatibility of the selected diluent used for infusion solutions, photostability studies to confirm the effect of light on the final product and transportation stability test at specified elevated storage conditions.

### Deficiencies in Module 3.2.P.1, description and composition of the FPP

There is 14% of deficiencies attributed to Module 3.2.P.1, Description and Composition of the FPP, from the whole 3.2.P section. The deficiencies in the section comprised requests for the potency adjustment calculation to be included. This equation clearly outlines the quantities required for the API depending on the assay of the API batches used. It also factors the water content present in the API and corrects to provide the acceptable quantity to be used. This should be included as a footnote under the composition table in 3.2.P.1. The other common deficiency in this section was on the indication of the polymorphic form used. The FPP manufacturer has to include the type of polymorphic form used in the batch formula as well as studies conducted to confirm the polymorphic form. They are required to provide the physico-chemical properties of the API in Module 3.2.P.2, pharmaceutical development, which will include polymorphic form investigation, particle size distribution and solubility. It should be noted that these parameters are not critical and may not be controlled by the final product manufacturer if the manufacturing process employs the following techniques which enhance the solubility as a result of the formation of the amorphous form of the product:

- Complete dissolution of the API in a diluent—results in the formation of an amorphous form [35].
- Hot melt extrusion which forms a solid dispersion of the API resulting in the formation of an amorphous polymer with enhanced solubility and bioavailability [36, 37].

The most common deficiency witnessed from sterile products in this section is on the request to include the pressure source used for filtration in the batch formula or composition list. The pressure source commonly used is nitrogen gas. It is also imperative that the pressure source used be sterile, this can be indicated in Module 3.2.P.4.

### Deficiencies in Module 3.2.P.7, container closure system of the FPP

The most common deficiencies in the section included the request for the following regarding the immediate container closure system:

- CoAs of the immediate container closure system (CCS),
- Identification, chemical nature and density of the container closure as well as specifications and the relevant control procedures,
- Colour, dimensions and thickness of the container closure system,
- The integrity for the heat seal bond strength (see Table 3).

Manufacturers are required to include the testing parameters used for the container closure system as well as analytical procedure used to do the test. Further description of the CCS is also frequently requested such as colour, dimensions and thickness. This needs to concur with the description in the PI and Patient Information Leaflet (PIL). This section also relates to Module 3.2.P.2.4 where developmental studies on the CCS should be conducted and the most common deficiency is that the manufacturers do not provide or poorly documenting the suitability of the container with the final product. This should include performance studies, suitability, compatibility and safety of the CCS. The common deficiency is frequently cited for sterile products in the section since compatibility studies with all components the final product is in contact with should be provided. For non-sterile products, a frequent response normally refers to the stability data provided in 3.2.P.8.3 or the confirmation that the reference product also uses the identical CCS. SAHPRA accepts these justifications.

### Comparison with other authorities

The reported deficiencies listed in Tables 3 and 4 have been compared with those published by other authorities and discussed below.

#### Comparison of deficiencies, SAHPRA vs USFDA

The USFDA published a four-part series on common deficiencies witnessed in the ANDA applications they received before 2010. Part 2–4 includes the common deficiencies found in the 3.2.P section of the CTD with Part 2 covering Module 3.2.P.1 and 3.2.P.4 on description, composition and excipients [5]. Part 3 covers Module 3.2.P.5 and 3.2.P.8 [6] while Part 4 covers the common deficiencies in Module 3.2.P.2/3 and 3.2.P.7, Manufacture and Container Closure System [7]. A quantitative comparison cannot be made since USFDA did not quantify the frequency of deficiencies. Some of the common deficiencies highlighted in 3.2.P.3 were on the in-process controls and tests (3.2.P.3.4, control of critical steps and intermediates) which is also 37% of deficiencies in the subsection by SAHPRA. Queries on granulation process was also reported to be significantly high and manufacturers were requested to provide a definitive quantitative end-point. A deficiency is included if no control or justification is provided by the applicant and the sole control proposed is a subjective, visual observation. For high shear processes, suitable controls may be related to the change in power consumption with respect to the granulation equipment (e.g., amperage). For fluid bed processes, moisture content can be a suitable control for end-point of the desired granules [7]. There were 5.9% of the deficiencies in the subsection requesting this by SAHPRA. For sterile products, the reported common deficiency was on excess fill volume and studies on extractable volume. A justification should be provided under manufacturing development based on data of multiple containers demonstrating that the intended volume can be extracted. Large overfills exceeding the required limit according to the USP 1151 general chapter [37, 38], should be appropriately justified as this may pose potential safety concerns. There were 9.6% of these deficiencies reported by SAHPRA for the applicable dosage forms. The most prevalent deficiency from Part 3 was on the control of the final product, specifications (3.2.P.5.1) which is also one of the highest common deficiency observed by SAHPRA at 58% in the subsection. The reported deficiencies are confirmed to be similar to those included in this study by SAHPRA.

#### Comparison of deficiencies, SAHPRA vs TFDA

A report by TDFA was made for applications submitted between June 2011 and May 2012 [8]. Deficiencies in the specification of the final product were the most prevalent in the final quality assessment reports. Issues regarding the specification of the final product were mainly related to the test item, related substances, or degradation products [8]. The second deficiency was for the validation of analytical procedures and mainly related to the validation for related substances/degradation products. The issues were mainly about the inadequate range/linearity and incomplete information about the characteristics (specificity, accuracy, precision, etc.) [8]. These deficiencies comprised 46% of subsection Module 3.2.P.5.3 for SAHPRA submissions. The other deficiency witnessed was regarding the manufacturing process which included inappropriate overages applied, an unjustified change in the manufacturing process, unclarified batch sizes, and others. These are similar to those reported by SAHPRA as seen from Tables 3 and 4 above. The top five deficiencies reported by SAHPRA are very similar to those reported by the TFDA (Table 5).

**Table 5:** Comparison of the top five common deficiencies from the five regulatory bodies listed below

SAHPRA^#^	TFDA	USFDA*	EMA	WHOPQTm
3.2.P.5.1	3.2.P.5.1	3.2.P.3.3	3.2.P.5	3.2.P.3
3.2.P.3.3	3.2.P.5.3	3.2.P.5.1	3.2.P.3	3.2.P.4
3.2.P.1	3.2.P.3.3	3.2.P.8	3.2.P.2	3.2.P.5
3.2.P.8.1/3	3.2.P.3.4	3.2.P.2.2	3.2.P.8	3.2.P.8
3.2.P.7	3.2.P.6	3.2.P.4	3.2.P.4	3.2.P.7

*USFDA did not report on the deficiency quantitatively

^#^Sequence included is for non-sterile products, the sequence is different for sterile products. Modules: 3.2.P.1 Composition and Description, 3.2.P.2 Pharmaceutical Development, 3.2.P.3.3 Description of the Manufacturing Process, 3.2.P.3.5 Process Validation or Evaluation, 3.2.P.8 Stability Data, 3.2.P.2.2 Pharmaceutical Development, 3.2.P.5.1 Specifications, 3.2.P.4 Control of the IPIs, 3.2.P.7 Container Closure System (see Table 2 for further descriptions)

#### Comparison of deficiencies, SAHPRA vs EMA

The study by the EMA was conducted on applications finalised by the CHMP, during 12 consecutive plenary meetings held in 2007 and 2008. The concerns raised by the Committee were on control of FPP (32% for 3.2.P.5.1), followed by concerns on the manufacturing (21% for 3.2.P.3), product development (17% for 3.2.P.2) and stability (17% for 3.2.P.8) [9]. This is similarly observed by SAHPRA as shown in Table 5, which compares the frequent deficiencies with what other authorities and organisations reported.

With respect to stability (3.2.P.8), 32% of concerns were regarding the lack of data submitted by the applicant to substantiate the proposed shelf-life of the FPP. For pharmaceutical development (3.2.P.2), 16% of concerns had to do with the results from comparative in vitro studies (for example the dissolution) or comparative in vivo studies (e.g., bioequivalence) requiring further discussion as well as a lack of information on the discriminatory power of dissolution method used [9]. These deficiencies were also observed by SAHPRA in the respective sections. The EMA also published a recent study reporting on common deficiencies witnessed for biosimilar submissions [15] Although these are different to orthodox medicines with respect to the API synthesis in most cases, there is similarity of these products with sterile products since most biosimilars are sterile. There were a number of similar deficiencies reported with those reported by SAHPRA. The deficiencies are; variety of media fill validation issues, validation of depyrogenation of glass vials and hold time validation issues in 3.2.P.3.5 (47% in the section), filter material and filter pore size not included in 3.2.P.3.3, lyophilisation conditions of the cycle used not indicated in 3.2.P.3.3 (28%) and compatibility studies of the FPP with the equipment not indicated in 3.2.P.2.4 (17%) [16]. Table 4 on the additional sterile product deficiencies also highlights these in the respective sections thereby confirming similarity.

#### Comparison of deficiencies, SAHPRA vs WHOPQTm

The WHOPQTm published FPP deficiencies observed in applications submitted between April 2007 and December 2010. The deficiencies reported were on missing executed and blank manufacturing records (BMRs), inadequate description of equipment, process parameters and end-point determination, inadequate description of sterile processes, unsatisfactory in-process tests and their frequency or acceptability of intermediate product specification, for Module 3.2.P.3 [3]. All the above have also been requested by SAHPRA as observed in Tables 3, 4 and 5. Previously, SAHPRA only requested the BMRs and packaging records when the need arose from the evaluations since they were the principle requirement during inspections. However, this condition was amended in 2020 by SAHPRA and is now a requirement during evaluations. Inadequate or poorly defined end-point for wet granulation process was another common deficiency as well as hold time related deficiencies from the guidance document [10]. These were also observed by SAHPRA and discussed in previous sections.

## Conclusion

The main objective of this study was to provide a comprehensive list of common deficiencies encountered by SAHPRA from the submitted 3.2.P section of CTD dossiers. The issues raised stem from product development, production and control of FPPs. The list is aimed at assisting manufacturers and applicants who submit future products to anticipate and avoid common pitfalls in regulatory affairs. Thus, as a result, this study will help pharmaceutical companies and manufacturers in reducing unnecessary and avoidable delays in the registration of these products to the benefit of accelerated access of medicines to patients. Comparisons with other regulatory authorities showed that other international regulatory agencies also observe similar common deficiencies as SAHPRA. This confirms the similarity in the extent of scientific assessments by the authorities, thus ensuring that quality, safe and efficacious medicines is available to patients.

### Limitations and future work

The study could not be conducted for applications finalised between 2018–2020 due to the following: the authority transitioned from the Medicine Control Council (MCC) to SAHPRA in 2018. In that time, SAHPRA staff continued to be housed in Civitas building in Pretoria with the National Department of Health employees. From April 2018, the department employees working in the Civitas building embarked on a protest action because of concerns about working conditions in the building. In the medium term, SAHPRA as a section 3A public entity, moved into new premises at the end of 2018. In addition, a backlog project was initiated in 2020, which required SAHPRA evaluators to implement, induct and train new evaluators involved in the project. As a result, information for 2018–2020 is not included in this study due to the disruptions caused by the protesting action, the move to the new premises and the initiation of the backlog project.

Further investigations will be conducted on other sections within the CTD to provide additional assistance in informing manufacturers and research organisations partaking in pharmaceutical development with the intent to obtain approval/registration from regulatory authorities.

## Data Availability

Data not available due to privacy and confidentiality restrictions.
